# Response Surface Methods Used for Optimization of Abrasive Waterjet Machining of the Stainless Steel X2 CrNiMo 17-12-2

**DOI:** 10.3390/ma14102475

**Published:** 2021-05-11

**Authors:** Andrea Deaconescu, Tudor Deaconescu

**Affiliations:** Department of Industrial Engineering and Management, Transilvania University of Brasov, 500036 Brasov, Romania; deacon@unitbv.ro

**Keywords:** abrasive waterjet machining, stainless steel, traverse speed, waterjet pressure, stand-off distance, abrasive grit size

## Abstract

Abrasive waterjet machining (AWJM) has a particularly high potential for the machining of stainless steels. One of the main optimization objectives of the machining of X2 CrNiMo 17-12-2 stainless steel is obtaining a minimal surface roughness. This entails selecting an optimum configuration of the main influencing factors of the machining process. Optimization of the machining system was achieved by intervening on four selected input quantities (traverse speed, waterjet pressure, stand-off distance, and grit size), with three set points considered for each. The effects of modifying the set-points of each input parameter on the surface roughness were studied. By means of response surface methodology (RSM) the combination of factor set points was determined that ensures a minimum roughness of the machined surface. The main benefit of RSM is the reduced time needed for experimenting.

## 1. Introduction

The applicability of stainless steels extends to industries like aerospace and defense, automotive, and chemical, due to its special properties like high resistance to corrosion and heat, strength, durability, low maintenance, and high hardness. Machining stainless steel by conventional methods is, however, difficult and costly, as it requires expensive tools with a modified geometry or large quantities of cooling fluids. Additionally, in the conventional cutting of stainless steels, the occurrence of vibrations has to be prevented, as these are responsible for tool edge deterioration (chipping). Because of such problems, alternative methods of machining stainless steels are called for, one of these being abrasive waterjet machining (AWJM). AWJM consists in the cutting—scratching—plastic deformation of the machined surface by a three-phase mixture of water drops (liquid), abrasive grains (solid), and air (gas) that impacts the workpiece at high speed. Material is removed by destroying the integrity of a workpiece surface under the dynamic action of erosion agents materialized in a coherent jet propelled at supersonic speed. Erosion is initiated by the kinetic energy of the fluid in motion [1,2].

Abrasive waterjet machining is generally used for hard materials in order to ensure high dimensional, form, and position accuracy of the machined surfaces. Further important benefits of AWJM are the quality of the obtained surfaces, as in certain situations a roughness of 10^−10^ m can be obtained, and the structure of the material layers that are machined or adjacent to the cutting area, as these are not or little affected by the action of the erosive agent [3]. Abrasive waterjet cutting has both advantages and challenges. Advantages include the wide range of machinable materials, low machining cost, high flexibility, easy programming and good productivity of processing, and conservation of material properties due to the low machining temperature. Specific challenges are the relatively low dimensional precision and some defects of the machined surface (striations, mini-craters, high roughness).

High pressure abrasive jet machining is increasingly deployed in industry. The 2019–2027 waterjet cutting machines market research report issued by Fortune Business Insights estimates an increase of sales from $1036.9 million in 2019 to $1675.1 million in 2027 [4]. With the global adoption of stringent environmental norms and standards, an increased demand for such cutting machines is to be expected. These machines release no hazardous materials or vapors and do not require any noxious gases or liquids while conducting the cutting process. The market growth will be also influenced by the addition of new software intelligence (CAD/CAM/Control software), the evolvement of 3D cutting, and the introduction of robotized machining [4].

Abrasive waterjet machining is an emerging technology that enables the machining of practically all materials [5]. Machining is possible of parts made from carbon and stainless steel [6], from composite materials [7,8,9,10], glass and marble [11], and so on. Based on the application, the cut-off thickness of materials can be typically 100 mm in stainless steel, 120 mm in aluminum, 100 mm in glass, and 140 mm in stone [12]. Complex cutting is required for turbines, blades, spares, and flanges in industries such as aerospace and defense, automotive, and energy and power. To achieve efficiency and productivity at every cutting process in real-time, robotic solutions are deployed [4].

Erosion-based machining systems using abrasive waterjets are extremely complex, as the process is influenced by a large number of factors. All physical phenomena occurring in the cutting area at very high pressures generate stochastic responses that require probabilistic and statistical calculations. Given the complexity of the phenomena occurring simultaneously during erosion, models are used in order to describe the machining process as closely to reality as possible. The modeling of abrasive waterjet machining aims at providing the system’s responses (such as material removal rate (MRR), surface roughness (Ra), depth of cut) based on input factors like waterjet pressure, abrasive mass flow rate, traverse speed, stand-off distance, and so on. The studies published by numerous researchers have proposed a number of such models developed by finite element analysis (FEA) or computational fluid dynamics (CFD) simulations. Other models are analytical, based on differential equations or statistical modeling, and supported by experimental results [5].

Because of the multitude of factors that influence AWJM, the studies conducted on this machining procedure concern only specific cases. Further on, research results found in literature are presented where statistical analysis methods were used for the processing of the experimental data. Thus, in [13], Monkova et al. analyzed the influence of traverse speed, abrasive mass flow rate, angle of attack, and depth of cut on the surface roughness of titanium surfaces. The experimental research was based on a full factorial design. Selvan and Raju studied in [14] the influence of waterjet pressure, nozzle traverse speed, abrasive mass flow rate, and stand-off distance on the roughness of cast iron surfaces. The effects of these factors on surface roughness were studied based on the experimental results obtained by using Taguchi’s design of experiments.

In [15], Benardos and Vosniakos present various methodologies and practices employed for the prediction of surface roughness. The approaches are classified into those based on machining theory, experimental investigation, designed experiments, and artificial intelligence (AI). Kumaran et al. investigate in [16] the surface roughness obtained as a result of machining of two different carbon-fiber-reinforced plastics (CFRP) with an abrasive waterjet machine. The experiments were conducted based on an L27 orthogonal array by varying the set-points of process parameters such as waterjet pressure, traverse speed, and stand-off distance. Regression models were developed to predict the correlation between the input parameters and the surface roughness for each composite. In [17], Kumaran et al. developed an adaptive neuro–fuzzy inference system (ANFIS) model for determining the surface roughness during machining of multidirectional woven fabric carbon-fiber-reinforced plastics (CFRP) using abrasive waterjet machining. Three variable input parameters—waterjet pressure, traverse speed, and stand-off distance—were selected to assess the roughness of the CFRP.

Because of the large number of input parameters that influence the output quantities of the abrasive waterjet machining system, most researchers turned to Taguchi’s approach for experimenting. The method allows the study of all parameters by a minimum of experimental runs. It replaces full factorial experiments by equally relevant, but less expensive and swifter, partial factorial experiments.

In [18], Kechagias et al. discuss a study based on Taguchi optimization. The considered input factors of the machining system are the geometry of the jet orifice and its distance to the machined surface, the magnitude of the material layer, and the traverse speed. The optimized output of the machined material (transformation-induced plasticity (TRIP) class steel) was surface quality expressed by two criteria, namely, roughness and width of the cut. Abrasive jet machining optimized by Taguchi’s methods can also be found in a paper published by Azmir et al. [19]. The controllable parameters of the process are matched against the targeted response, namely surface roughness and the gradient of the cut depth. In this study, the machined material was a composite plastic known as aramid fiber-reinforced polymer (AFRP). The analysis identified the traverse speed as responsible for the most significant impact on the response parameters. The authors find that both quality criteria are improved, meaning the smaller roughness values and more vertical kerf walls are obtained for discharging the abrasive jet from a smaller distance at a greater pressure and smaller traverse speed.

Another method besides Taguchi optimization is the response surface methodology (RSM) used for adjusting controllable input factors, such as to obtain the targeted responses of the machining system. In [20], the authors discuss waterjet cutting of materials consisting of biodegradable polymers bound by natural components. Again, the main impacting controllable factors are considered the hydraulic pressure, the traverse (feed) speed, and the stand-off distance. The optimized output factors in this application were the quality of the surface expressed by its smoothness (roughness) and the duration of machining. Another paper reporting RSM is [21], where Yue et al. addressed the machining of a specific class of ceramics having a high aluminum oxide concentration. The quality criterion to be improved was the MRR, and the input factors adjusted correspondingly to the optimization results included jet kinematic characteristics (feed rate and pressure), jet composition dynamics, and machine settings (workpiece speed and inclination of the nozzle). An interesting contribution to waterjet machining is brought by Sutowska et al. [22], who attempt to prove the influence of cutting trajectory curve radii on fragile workpieces, namely on the smoothness of the kerf. The authors conclude that soda-lime-silicate galls should be cut by jet following spiral trajectories. AWJM machining of stainless steels was less studied. In [23], Arola and Ramulu focus on the material characteristics rather than on machining parameters. They work with a number of different types of steel, including type 304, to demonstrate how the damage to the surface caused by abrasive jet machining fluctuates by material. The same steel was studied also by Akkurt et al. in [24]. One of the input factors considered was the traverse speed. The authors reach the conclusion that lower set-points for this factor, while yielding a smoother surface for small values of material layer thickness, will have the opposite effect on thick workpieces. Further papers addressing stainless steel machinability by AWJM are [25,26,27].

The literature review yielded the fact that no general computational relationships have been identified to link the various parameters that influence the results of machining. The published studies present the values obtained for output quantities like roughness, duration of machining, the thickness of the cut-off material layer, and material removal strictly for certain concrete cases—results that cannot be generalized. Another conclusion of the literature review concerns the fact that waterjet machining of stainless steels has been insufficiently studied, and that further research is call for in order to determine an optimum configuration of the work parameters. Starting from these considerations, this paper discusses the experimental investigation of the cutting performance of AWJM of X2 CrNiMo 17-12-2 austenitic stainless steel. The research presented in this paper investigates the influence of four parameters, namely, waterjet pressure, traverse speed, grit size, and stand-off distance, on the surface finish of austenitic steel. Response surface methodology (RSM) is applied to analyze the system’s responses. The paper is structured as follows: The Introduction is followed by Section 2, which introduces the main work parameters that influence the machining process. Section 3 presents the work method and deployed equipment. Section 4 discusses the results and is followed by the final section of conclusions.

## 2. Parameters of the Abrasive Waterjet Process

It has been argued [28] that AJM can be viewed as a form of abrasive wear, considering the small mass of cut-off material. Such wear, however, is just one of the components of a more complex processes that includes also corrosion, material failure, and adherence phenomena. Consequently, the impact of a series of factors on the removal of material has to be included in a wider analysis of AWJM.

**Figure 1:**
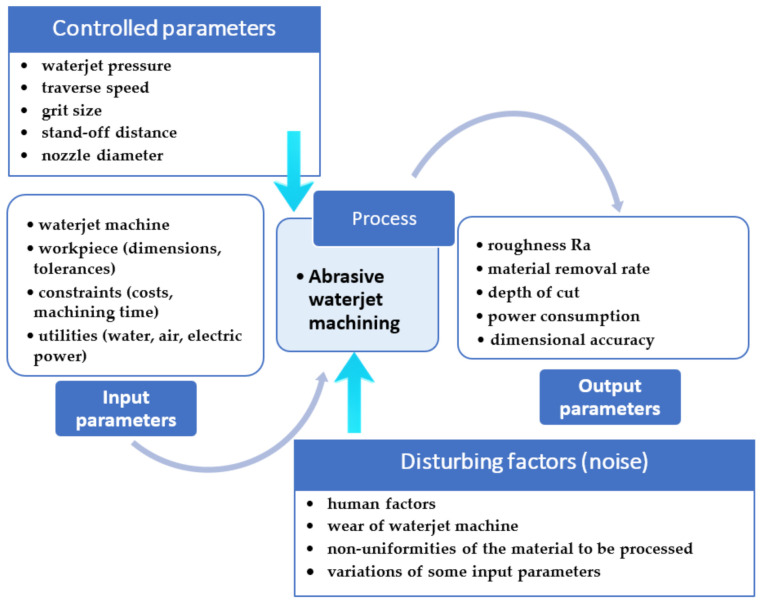
AWJM parametric diagram.

AWJM is an open system with inputs set according to the technical possibilities, and outputs that are finished parts with precise dimensions and technical specifications. The machining process is subject to factors that can be partly controlled, and others that are uncontrollable. All influencing parameters are interdependent, and the modification of one factor can lead to many possible results, with an evolution that is not linear. This is determined by the effects that modifying the set-point of one parameter has on other parameters that depend on the former. A finer graining, for example, does not necessarily yield a better surface roughness (abrasive graining influences mass flow, which, in turn, can cause an increase of roughness) [29]. Figure 1 presents the AWJM parametric diagram.

The input quantities of the system are technical and economic and can be grouped into fixed input signals (the material properties of the part, network feed specifics, certain parameters of the equipment—number of axles, machining precision) and into adjustable, controllable ones (like pressure, traverse speed, stand-off distance, etc.). The influence of the fixed signals on the variability of the result is considered zero, and only the variable and controllable signals are used for process optimization.

The output quantities are the values that are expected upon running the open machining system consequently to configuring the controlled input factors in an optimized manner. Noise is the action of a disturbing, uncontrollable factor that triggers unpredictable responses, generally considered random events. Each noise has a small influence on the system, or else it has to become a controlled quantity.

Figure 1 illustrates that the machining process is influenced by a very large number of parameters, each of which can have several set-points. Obtaining an optimum machining result entails the testing of all possible combinations of influencing factor set-points. As this is nearly impossible due to a large amount of work, time, and material involved, a rapid modality has to be identified for identifying the optimum combination of input factor set-points that yield a satisfying result of the machining process that satisfies both technical and economic requirements.

The method known as design of experiments (DoE) lends itself to solving this optimization problem. DoE is a planned approach to determining cause and effect relationships. It can be applied to any process with measurable inputs and outputs [30]. Planning experiments for analyzing the AWJM process can be made by: i. random testing, ii. testing the influence of a single factor at one time, iii. testing all possible combinations of factor set-points, iv. fractioned testing or testing by means of orthogonal arrays of experiments (Taguchi’s method), or v. by applying response surface methodology (RSM). Selecting one or the other of these experimenting techniques depends on the number of influencing parameters considered and on the required accuracy of the engineering analysis.

The study discussed in the paper was based on response surface methodology (RSM). From the multitude of parameters that influence the erosive machining process, based on the relevant engineering experience of the authors, four main impacting parameters were identified: the traverse speed Vt (mm/min), waterjet pressure p (MPa), stand-off distance h (mm), and grit size G (Mesh). The considered output quantity was the surface roughness Ra (μm).

## 3. Materials and Methods

### 3.1. Materials

The experiments were aimed at optimizing the machining of X2 CrNiMo 17-12-2 austenitic stainless steel (equivalent to 316L AISI Standard). This material has medium to poor machinability and a very good corrosion resistance, and is mainly used in vehicle construction, and the chemical industry, food industry, medical/pharmaceutical industry, and building industry. The chemical composition (wt.%) according to DIN of this steel is C ≤ 0.03%, Cr 16.5–18.5%, Ni 10.5–13.0%, Mo 2.0–2.5%.

### 3.2. Equipment

Machining consisted of cutting a 30 mm diameter bar on a Maxiem 1530 abrasive waterjet cutting machine (OMAX, Seattle, WA, USA) that is endowed with Intelli-Max software developed by Omax, and includes two packages: Intelli-Max Layout (the graphic part) and Intelli-Max Make (the execution part). Intelli-Max Make allows the configuring of the main factors that influence the machining process, as well as entering the machining data: material thickness, material machinability, cutter head trajectory control, stand-off distance to the material, and so on.

The input data used in this study were the traverse speed Vt (mm/min), waterjet pressure p (MPa), stand-off distance h (mm), and grit size G (Mesh). The expected result was to define a technological process that improves the quality of the machined surface expressed by its roughness Ra (µm). Each run was carried out one time, and for each one run, the surface roughness was measured 5 times. The surface roughness was measured at half of the workpiece thickness (15 mm). Thus, 5 measured results were obtained for each run. Subsequently the arithmetic mean of these 5 measured results was computed. The travel of the roughness sensor was of 4 mm, perpendicular to the direction of the abrasive jet. The factor representing the traverse speed was selected such as to still allow cutting at a minimum pressure, using a minimum diameter tube and an abrasive of smaller graining (grit size), while positioning the cutter head at maximum stand-off distance.

The roughness gauge used for measurements was TESA Rugosurf 10G, made by Tesa, Switzerland.

**Figure 2:**
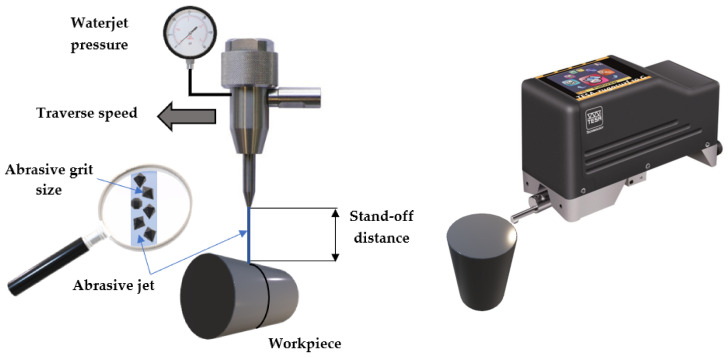
AWJM system.

Each configurable parameter (factor) was assigned 3 possible set-points (Table 1):

Figure 2 shows a schematic of the machining process, and the utilized roughness gauge.

### 3.3. Methods

In this study, the authors applied response surface methodology (RSM, Box–Behnken design) to the measured responses. RSM allows the plotting of a response graph by shape or a spatial representation [30]. RSM is underlain by DoE and deploys fractioned experimental arrays, that is, a small number of runs to isolate the influence of the various considered adjustable parameters of the machining process [31].

By feeding the values measured experimentally into the RSM instruments, a regression equation is generated that is subsequently used to determine optimum set-points for the controlled parameters such as to yield the desired values of the quality criterion to be optimized [32]. The mathematical model of RSM can be expressed as Equation (1):

(1) $$y=f\left(x_{1},x_{2}\right)+\varepsilon$$

where *ε* is the response error, and *f(x_1_, x_2_)* is the expected response. It can be noticed that this method entails analyzing the studied phenomenon by simultaneously modifying the set-points of only two work parameters (factors) while maintaining the others constant.

## 4. Results and Discussion

Applying response surface methodology (Box–Behnken design) meant maintaining two parameters constant in turns, at their median set-points, while modifying the rest of two parameters. In this work plan, the number of possible test variants is six.

In the first set of tests, machining was carried out at a stand-off distance set to 1.5 mm, using a mesh size #100 garnet abrasive. The other two work parameters were adjusted successively to the three possible predetermined set-points. The resulting array of experiments includes nine runs, each traverse speed set-point being combined with the three set-points of the waterjet pressure. Table 2 shows the measured results.

In Table 2, the standard deviation illustrates the statistical dispersion of the measured values. The values of the standard deviation are noticeably small, close to zero, and indicate a low uncertainty of the measurements.

**Figure 3:**
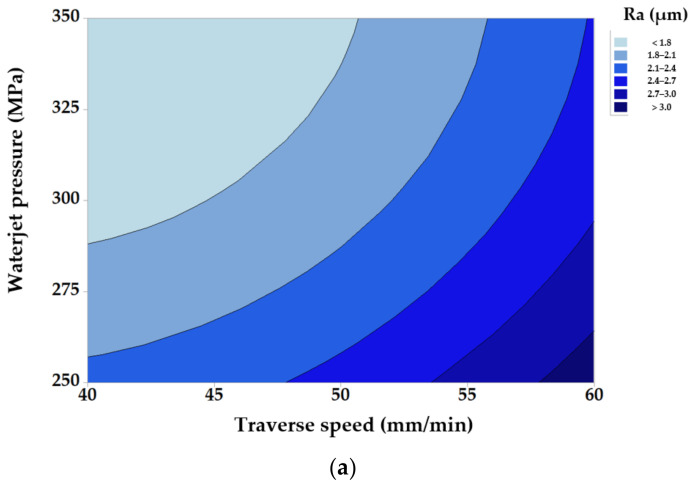

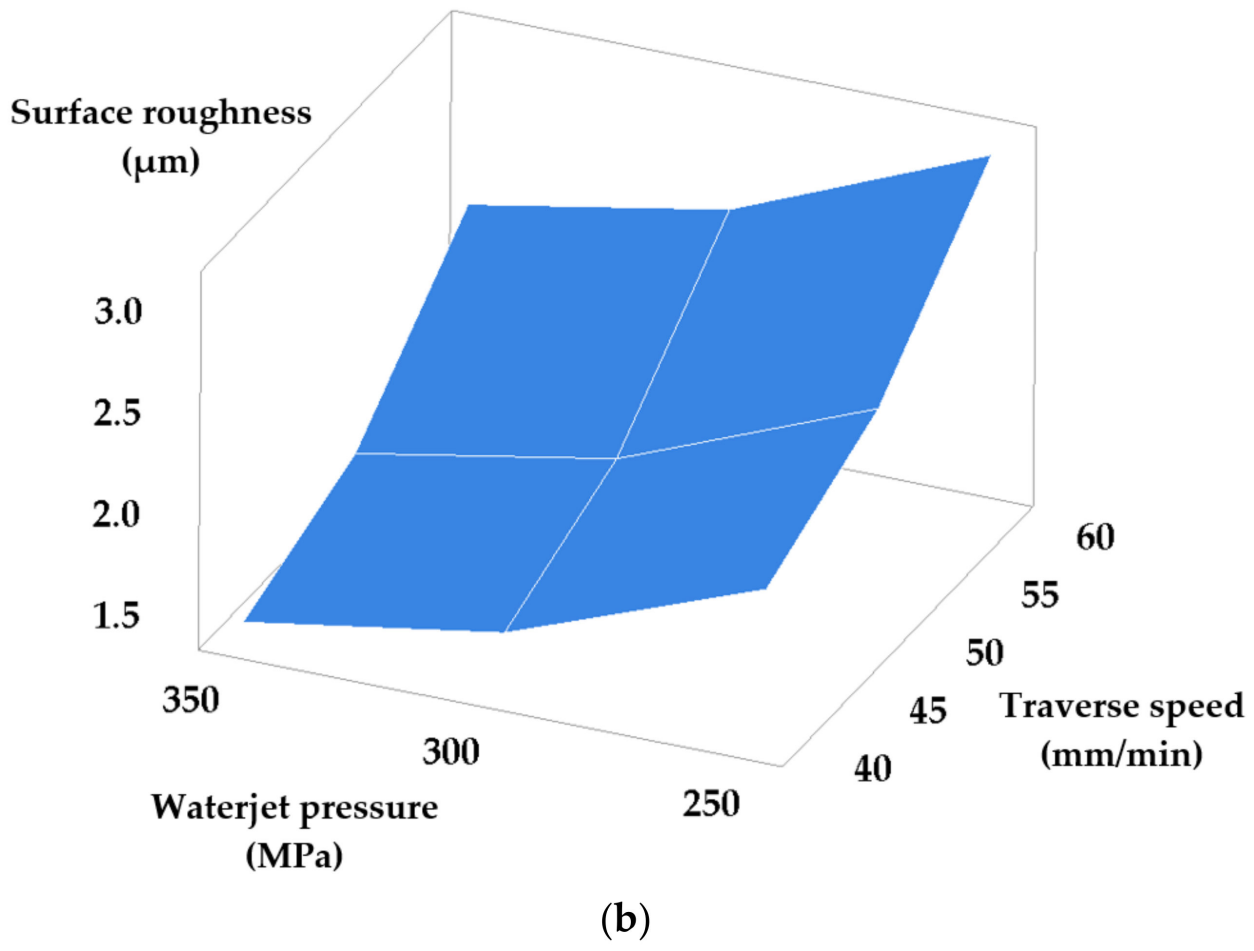
Roughness versus traverse speed and waterjet pressure set-point modification: (**a**) contour representation; (**b**) 3D response-surface.

Figure 3 illustrates the effects of modifying traverse speed and waterjet pressure set-points, while maintaining fixed values of the other parameters.

Each contour in Figure 3a represents a combination of input factors that produces a constant response. As the information yielded by the contour representation is less suggestive, only 3D representations will be shown for the following tests.

From Figure 3, it follows that decreasing water pressure simultaneously with increasing traverse speed has a negative influence on the quality of the machined surface (greater roughness). The obtained results can be explained as follows: a high pressure of the abrasive jet causes a higher jet speed, and implicitly, an increase of its kinetic energy. A high-kinetic-energy abrasive jet will remove material only by cutting and not by plastic deformation. This is favorable, as cutting leads to a better roughness, because it leaves finer traces on the machined surface, traces of smaller width and depth than plastic deformation.

It is known that machining with a low pressure, thus low speed and low energy jet—caused by jet–material interaction and mutual particle impact—determines the plastic deformation of the material along with material removal by cutting. Because such plastic deformation increases surface roughness, low pressure jets should be avoided. Experiments confirm this: diminishing waterjet pressure from 350 MPa to 250 MPa leads to a 0.8 µm increase in surface roughness.

Experiments revealed an increase of roughness with a greater traverse speed. A smaller traverse speed allows for more time for a larger number of abrasive particles to participate in cutting, and thus, a larger number of asperities is removed leading to a better surface quality.

For the analyzed data shown in Table 2, Equation (1) becomes:

(2) $$\mathrm{Ra}=1.998+0.04753\;\cdot \;V_{t}-0.0072\;\cdot \;p$$

**Figure 4:**
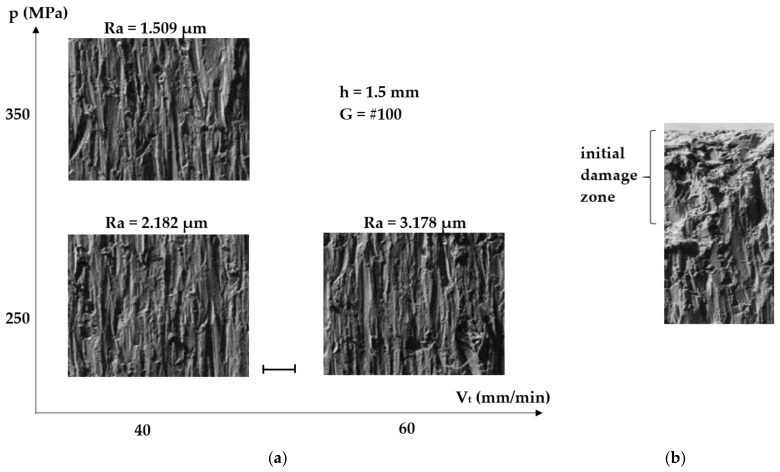
AWJ-machined surface: (**a**) traces; (**b**) initial damage area.

The microstructures of the cut surfaces were observed by scanning electron microscopy (SEM) for three sets of values of the traverse speed and the waterjet pressure (40 mm/min, 250 MPa; 40 mm/min, 350 MPa; and 60 mm/min, 250 MPa, respectively), as shown in Figure 4.

The images in Figure 4a show the areas where the roughness was measured, namely at half of the workpiece thickness (15 mm), while Figure 4b shows the impact area of the jet on the machined surface. The vertical traces left by the abrasive grain visible in Figure 4a have a higher density and are less deep at a small traverse speed (40 mm/min) and high pressure (350 MPa). From the quantitative viewpoint of the machining process addressed by the study, there are no significant visual differences between the three images shown in Figure 4a, representing the traces left by the abrasive for different values of the traverse speed and the waterjet pressure. Consequently, as the SEM images of the microstructure do not offer quantitative information, research was focused on measuring surface roughness and discussing only those values.

The image shown in Figure 4b corresponds to machining with a traverse speed of 40 mm/min and a waterjet pressure of 250 MPa. It can be noticed that up to a depth of about 200 µm, the surface layer is slightly damaged by plastic deformation caused by the strong impact of the abrasive jet. Such damage is inherent to this machining process and acceptable, as beyond this initial impact area the desired surface smoothness is obtained.

**Figure 5:**
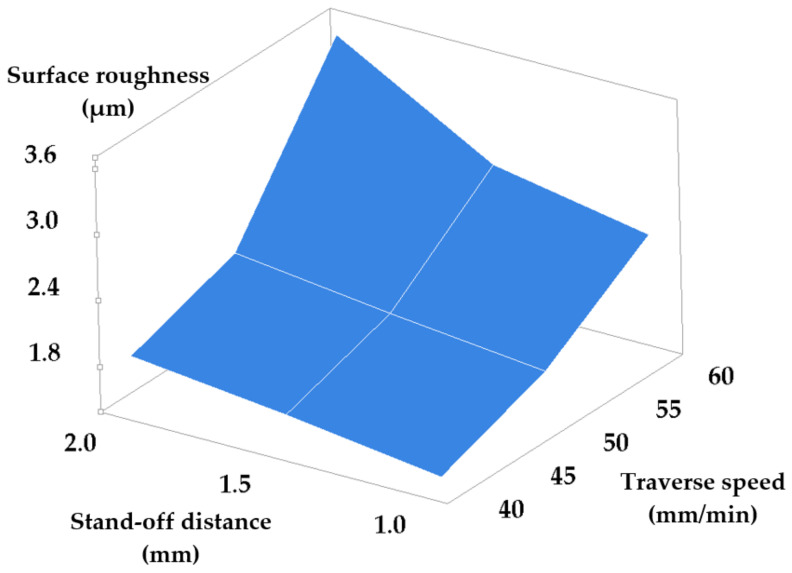
Roughness versus stand-off distance and traverse speed set-point modification.

**Figure 6:**
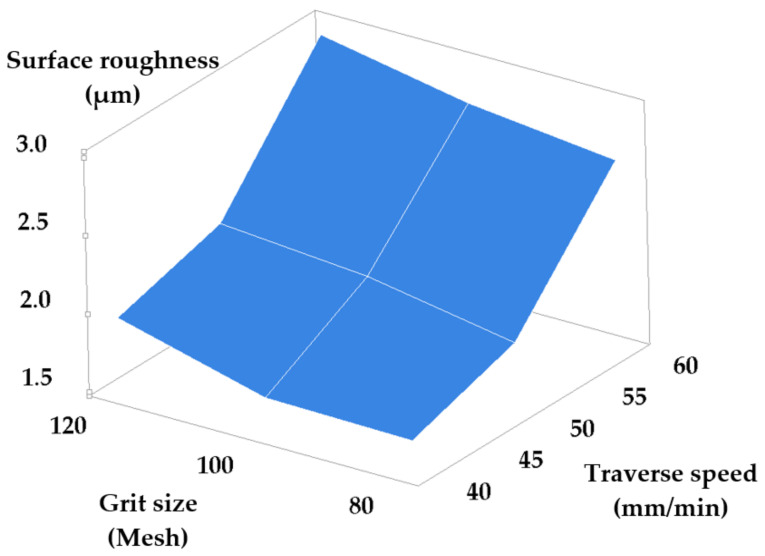
Roughness versus grit size and traverse speed set-point modification.

Table 3 and Table 4 present the results obtained consequently to machining while modifying the set-points of the traverse speed and stand-off distance, and of the traverse speed and grit size, respectively. The values of the standard deviation show a low measurement uncertainty. Figure 5 and Figure 6 show the dependencies of surface roughness in these two cases.

In this case, increasing traverse speed combined with increasing the stand-off distance causes a significant increase of roughness, thus damaging surface quality. This can be explained by the dependence of the kinetic energy of the abrasive jet in the cutting area on the stand-off distance—namely, a smaller stand-off distance maintains the coherence of the abrasive jet and consequently ensures its high kinetic energy at the impact on the surface. A high kinetic energy of the abrasive jet ensures a sufficient impact force such as to cut and remove the asperities of the machined surface. In addition, jet kinetic energy is higher for small stand-off distances, and consequently, penetration will be deeper and the kerf narrower.

Equation (3) follows from the analyzed data that are shown in Table 3:

(3) $$\mathrm{Ra}=-1.784+0.0626\;\cdot \;V_{t}+0.587\;\cdot \;h$$

A high traverse speed and an increased grit size also have a negative impact on the quality of the machined surfaces. Small values of the traverse speed are recommended in order to maximize the number of the effective abrasive grains that cut the surface. The obtained roughness is determined by the density of the traces left by the abrasive grains. Smaller size abrasive grains will remove thinner microchips from the material and cause finer traces on the surface. Thus, combining a low traverse speed with small grain size will determine a dense pattern of fine traces resulting in good surface roughness.

Equation (4), provided by the Minitab program based on the data in Table 4, represents an accurate description of the evolution of roughness versus the analyzed input parameters.

(4) $$\mathrm{Ra}=-1.026+0.05125\;\cdot \;V_{t}+0.00675\;\cdot \;G$$

**Figure 7:**
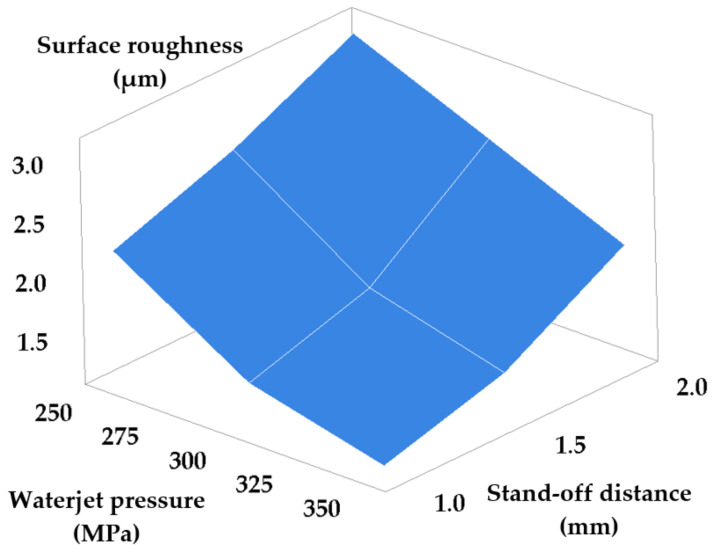
Roughness versus waterjet pressure and stand-off distance set-point modification.

**Figure 8:**
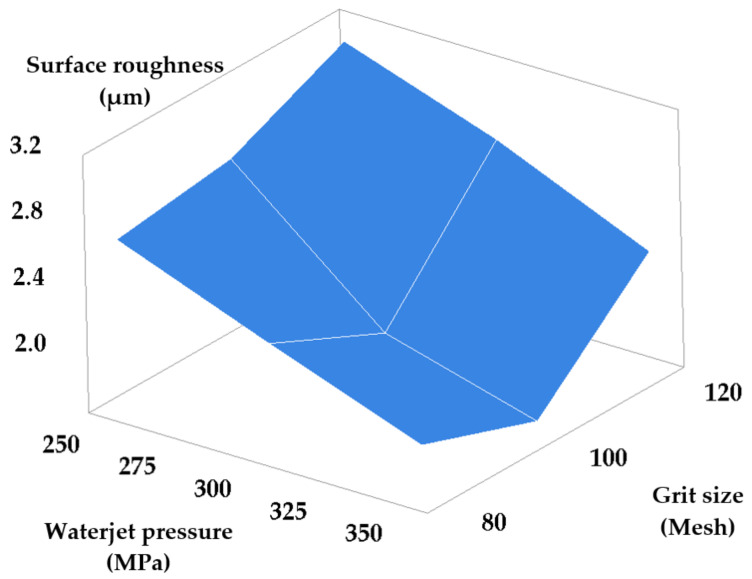
Roughness versus waterjet pressure and grit size set-point modification.

Another set of measurements entailed the simultaneous modification of waterjet pressure and stand-off distance set points, and of waterjet pressure and grit size set-points, respectively. The other two parameters were maintained at constant values. Table 5 and Table 6 present the results obtained consequently to machining while modifying the set-points of the waterjet pressure and stand-off distance, and of the waterjet pressure and grit size, respectively. Figure 7 and Figure 8 show the dependencies of machined surface roughness in these two cases.

The graph in Figure 7 yields the following conclusions: increasing work pressure at the same time with decreasing the stand-off distance between tube and workpiece ensures a smaller surface roughness, because, as discussed, a coherent abrasive jet of high kinetic energy is obtained, capable of ensuring the operation to be carried out by cutting rather than by plastic deformation. Consequently, a better surface quality will be obtained.

The following equation describes the variation of roughness depending on the values of the pair of parameters p and h.

(5) Ra=3.912−0.01021 · p+0.941 · h

The graph in Figure 8 yields the following conclusion: a better roughness is obtained for higher jet pressures and median grit size (mesh size #100). High jet pressure favors material removal by cutting rather than by plastic deformation, while a finer grit size yields a favorable high-density pattern of less deep traces.

Equation (6) describes the variation of roughness versus the considered pair of parameters (p and G):

(6) Ra=3.877−0.00826 · p+0.01034 · G

**Figure 9:**
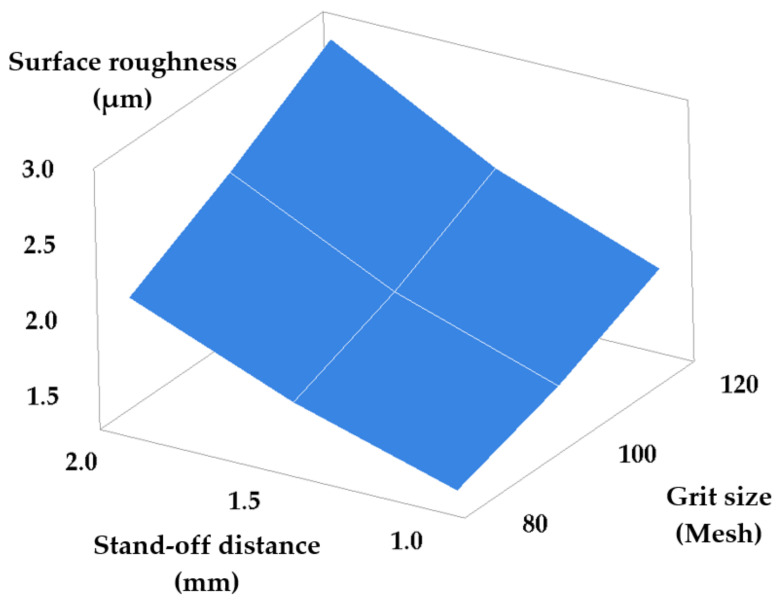
Roughness versus stand-off distance and grit size set-point modification.

The last possible combination requires modifying the stand-off distance and grit size, while traverse speed and waterjet pressure are kept constant. Table 7 and Figure 9 show the results obtained consequently to machining.

In Table 5, Table 6 and Table 7, the values of the standard deviation are noticeably small, and indicate a low uncertainty of the measurements.

Figure 9 shows that a better roughness is obtained for fine graining (small grit size) and a small stand-off distance. On one hand, as discussed, a small stand-off distance ensures a high kinetic energy of the abrasive jet that is consequently capable of machining only by cutting. On the other hand, small grit sizes cause a dense pattern of small-depth traces on the machined surface. Both these aspects contribute to obtaining small roughness values, that is, a good surface quality.

A good quality of the cut surface requires the optimum correlation of stand-off distance and abrasive grain size. A small stand-off distance calls for a fine grit, because small grains are easier removed from the cutting area. The efficiency of the jet could be diminished at small stand-off distances because of jams caused by large grains that collide with the fresh particles fed to the cutting area.

In this case the obtained roughness is described by the following equation:

(7) Ra=1.155+0.652 · h−0.004 · G

In order to achieve the objective of the study, namely minimizing the roughness of machined surfaces, all six graphs presented above were analyzed in parallel. The following recommendations emerged:

- the traverse speed has to be as small as possible (recommended value: 40 mm/min);
- the waterjet pressure has to be as high as possible (recommended value: 350 MPa);
- the stand-off distance has to be small (recommended value 1 mm);
- the surface roughness is less sensitive to the abrasive graining, and a mesh size of #80 or #100 is recommended.

From a quantitative viewpoint, the graphs reveal that the traverse speed has a major influence on the quality of the machined surface. For the analyzed interval of the traverse speed ranging from 40 to 60 mm/min, the surface roughness registered values between 1.514 μm and 3.576 μm. A further significant influence on surface roughness is that of the waterjet pressure. Thus, for the analyzed interval ranging from 250 to 350 MPa, the surface roughness registered values between 3.267 and 1.405 μm. The variation of the stand-off distance between 1 and 2 mm caused values of the surface roughness ranging from 1.405 μm to 3.576 μm. For grit sizes between 80 and 120 Mesh, the surface roughness varied between 1.461 μm and 3.114 μm.

These observations lead to a general conclusion, namely that a good surface quality is obtained by selecting a small traverse speed, a high jet pressure, a small stand-off distance, and fine grit sizes. The obtained results allowed setting an optimized configuration of the machining process such as to minimize surface roughness. Table 8 shows the optimum set points of the work parameters, determined by experiment. Based on these conclusions, a final machining process was performed, with work parameter set-points adjusted according to the recommendations presented above (Table 8). The surface roughness obtained under these circumstances was of 1.382 μm.

## 5. Conclusions

This paper presents a study of abrasive waterjet cutting, optimized by response surface methodology (Box–Behnken design). The aim of the study was to obtain minimum surface roughness consequently to cutting workpieces made from X2 CrNiMo 17-12-2 austenitic stainless steel. The novelty of this study consists in introducing response surface methodology as a useful instrument for optimizing AWJ machining of stainless steels, due its main benefit of reduced experimenting time.

The AWJM process is extremely complex, as it is subject to the influence of a multitude of parameters—controllable or not. Minimum surface roughness can be obtained by selecting an adequate combination of the input factor set-points. Four controllable input factors (waterjet pressure, traverse speed, stand-off distance, and abrasive grit size) were selected, and three set-points were considered for each.

Utilizing response surface methodology requires maintaining two parameters constant in turns, at their median set points, while modifying the other two parameters. The experiments conducted with the considered input parameters grouped into pairs proved that these work parameters directly impact surface roughness.

For the considered set-points of the input parameters, the surface roughness varied between 1.405 μm and 3.576 μm. It has been found that an increase in waterjet pressure is associated with a decrease in surface roughness. As regarding the other considered parameters, good surface quality is obtained by using small traverse speed and stand-off distance values, and medium to fine abrasive graining (grit size).

Reduced surface roughness could be obtained upon assigning the recommended optimum set-points to the input parameters, thus achieving the aim of the study. Thus, following the experiments the optimum set-points of the input parameters were established, and upon running the respective validation experiments, a very good roughness of 1.382 μm was obtained.

## Figures and Tables

**Table 1:** Configuration of the input parameter (factor) set-points.

No.	Traverse Speed Vt (mm/min)	Pressure p (MPa)	Stand-Off Distance h (mm)	Grit Size G (Mesh)
1	40	250	1	80
2	50	300	1.5	100
3	60	350	2	120

**Table 2:** Roughness values obtained upon adjusting traverse speed and water pressure.

Traverse Speed V_t_ (mm/min)	Pressure p (MPa)	Stand-Off Distance h (mm)	Grit Size G (Mesh)	Roughness R_a_ (µm)	Standard Deviation
40	250	1.5	100	2.182	0.0024
50	250			2.501	0.0025
60	250			3.178	0.0027
40	300			1.712	0.0043
50	300			1.998	0.0023
60	300			2.654	0.0022
40	350			1.509	0.0024
50	350			1.768	0.0027
60	350			2.423	0.0031

**Table 3:** Roughness values obtained upon adjusting traverse speed and stand-off distance.

Traverse Speed V_t_ (mm/min)	Pressure p (MPa)	Stand-Off Distance h (mm)	Grit Size G (Mesh)	Roughness R_a_ (µm)	Standard Deviation
40	300	1	100	1.514	0.0025
50		1		1.869	0.0032
60		1		2.504	0.0029
40		1.5		1.708	0.0034
50		1.5		2.023	0.0034
60		1.5		2.768	0.0027
40		2		1.871	0.0032
50		2		2.202	0.0032
60		2		3.576	0.0032

**Table 4:** Roughness values obtained upon adjusting traverse speed and grit size.

Traverse Speed V_t_ (mm/min)	Pressure p (MPa)	Stand-Off Distance h (mm)	Grit Size G (Mesh)	Roughness R_a_ (µm)	Standard Deviation
40	300	1.5	80	1.685	0.0039
50			80	1.908	0.0029
60			80	2.674	0.0034
40			100	1.702	0.0023
50			100	2.076	0.0034
60			100	2.782	0.0023
40			120	1.957	0.0025
50			120	2.157	0.0034
60			120	2.963	0.0034

**Table 5:** Roughness values obtained upon adjusting waterjet pressure and stand-off distance.

Traverse Speed V_t_ (mm/min)	Pressure p (MPa)	Stand-Off Distance h (mm)	Grit Size G (Mesh)	Roughness R_a_ (µm)	Standard Deviation
50	250	1	100	2.412	0.0021
	300	1		1.699	0.0029
	350	1		1.405	0.0023
	250	1.5		2.777	0.0023
	300	1.5		2.012	0.0024
	350	1.5		1.699	0.0034
	250	2		3.267	0.0025
	300	2		2.782	0.0022
	350	2		2.289	0.0019

**Table 6:** Roughness values obtained upon adjusting waterjet pressure and grit size.

Traverse Speed V_t_ (mm/min)	Pressure p (MPa)	Stand-Off Distance h (mm)	Grit Size G (Mesh)	Roughness R_a_ (µm)	Standard Deviation
50	250	1.5	80	2.703	0.0023
	300		80	2.345	0.0023
	350		80	2.001	0.0037
	250		100	2.798	0.0046
	300		100	2.015	0.0021
	350		100	1.752	0.0016
	250		120	3.114	0.0021
	300		120	2.791	0.0031
	350		120	2.385	0.0023

**Table 7:** Roughness values obtained upon adjusting stand-off distance and grit size.

Traverse Speed V_t_ (mm/min)	Pressure p (MPa)	Stand-Off Distance h (mm)	Grit Size G (Mesh)	Roughness R_a_ (µm)	Standard Deviation
50	300	1	80	1.461	0.0016
		1.5	80	1.783	0.0025
		2	80	2.215	0.0023
		1	100	1.691	0.0023
		1.5	100	2.053	0.0035
		2	100	2.582	0.0034
		1	120	2.006	0.0022
		1.5	120	2.409	0.0026
		2	120	3.001	0.0035

**Table 8:** Roughness values obtained consequently to machining with the work parameters adjusted to the optimum recommended values.

Traverse Speed V_t_ (mm/min)	Pressure p (MPa)	Stand-Off Distance h (mm)	Grit Size G (Mesh)	Roughness R_a_ (µm)
40	350	1	100	1.382

## Data Availability

The data presented in this study are available in this article and in [28,29].

## Nomenclature

AWJM	abrasive waterjet machining
V_t_	traverse speed [mm/min]
p	waterjet pressure [MPa]
h	stand-off distance [mm]
G	grit size [Mesh]
Ra	surface roughness [μm]
RMS	Response Surface Methodology
